# Dienogest as a Maintenance Treatment for Endometriosis Following Surgery: A Systematic Review and Meta-Analysis

**DOI:** 10.3389/fmed.2021.652505

**Published:** 2021-04-07

**Authors:** Yijun Liu, Han Gong, Jinhai Gou, Xinghui Liu, Zhengyu Li

**Affiliations:** ^1^Department of Gynecology and Obstetrics, West China Second University Hospital, Sichuan University, Chengdu, China; ^2^Key Laboratory of Obstetrics and Gynecologic and Pediatric Diseases and Birth Defects of Ministry of Education, West China Second Hospital, Sichuan University, Chengdu, China

**Keywords:** dienogest, endometriosis, surgery, maintenance treatment, meta-analysis

## Abstract

This study aimed to comprehensively assess the value of Dienogest (DNG) as a maintenance treatment following conservative surgery for endometriosis in terms of the outcomes of disease and pregnancy. We searched for relevant studies and trials up to November 2020 from PubMed, Cochrane Library, Medline, and EMBASE databases as well as the Web of Science. Patients who received DNG maintenance treatment were compared to those who received other treatments (OT), including the levonorgestrel-releasing intrauterine system (LNG-IUS) and gonadotropin-releasing hormone analogs (GnRH-a), or non-treatment (NT). The primary outcomes were disease recurrence and pregnancy rates. Eleven studies were included in this meta-analysis. The pooled analysis indicated that DNG maintenance treatment was associated with a lower rate of disease recurrence. A significant difference was observed in DNG maintenance treatment compared with NT, but not with OT, in the pregnancy rates postoperatively. Moreover, DNG maintenance treatment was related to a significant increase in vaginal bleeding and weight gain. DNG can be recommended as a maintenance treatment for patients with endometriosis to decrease the rates of disease recurrence following conservative surgery. However, DNG maintenance treatment has no advantage in improving pregnancy rates compared to OT.

## Introduction

Endometriosis is estimated to affect 10–15% of women of childbearing age and as much as 15–30% of those with primary or secondary infertility (1, 2). It is an estrogen-dependent disease, wherein the endometrial tissue deposits outside the uterus, typically leading to chronic pelvic pain, dysmenorrhea, dyspareunia, fatigue, and infertility. Initial management of the disease revolves around conservative surgery, preserving the patients' fertility, removal of ectopic lesions, and relieving the symptoms of discomfort. Laparoscopic surgeries, as the first choice, are characterized by less trauma, quick postoperative recovery, and a simple operative procedure. However, treatment is challenging in patients with a deep invasion of endometriotic lesions and severe adhesions to the pelvic cavity, as it is difficult to ensure that they are completely removed and, therefore, easily recur after surgery. Notably, the rate of recurrence in endometriosis ranges between 30 and 50% (3, 4). In addition, numerous studies exist on the recurrence of endometriosis, which, in some instances, leads to malignant transformation to ovarian cancer (5) and reoperation. Moreover, reoperation after the recurrence of endometriosis, often damages the remaining ovarian function, causes menstrual abnormalities, amenorrhea, infertility, and an increase in costs; hence, open surgery and definite surgery (e.g., total abdominal hysterectomy bilateral salpingo-oophorectomy) may be required. Consequently, continuous consolidation and long-term medication management following conservative surgery are crucial.

Dienogest (DNG) is a selective progestin that only binds to progesterone receptors and is a unique fourth-generation synthetic progestogen, recommended by multinational guidelines as the first-line drug for the long-term management of endometriosis. It is characterized by good tolerance, anti-androgen activity, moderate inhibition of the hypothalamic-pituitary-ovarian (H-P-O) axis, moderate reduction of estrogen levels, and minimal impact on estrogen, sugar, salt, and lipid metabolism (6). Compared to gonadotropin-releasing hormone analogs (GnRH-a) treatment, DNG maintains the levels of estradiol within the estrogen window dose for the treatment of endometriosis. This implies that the amount of estrogen in the body is maintained at a level that does not stimulate ectopic endometrial growth, without causing perimenopausal symptoms and loss of bone density, and simultaneously reduces the side effects without affecting the treatment effectiveness. Besides, the levonorgestrel-releasing intrauterine system (LNG-IUS) is not suitable for women with fertility requirements; however, endometriosis is more likely to occur in women of childbearing age who have fertility requirements.

Studies on the significance of DNG as a post-operative maintenance treatment compared to other treatments (OT) or no treatment (NT) remain scarce. Moreover, there are inconsistent conclusions among existing studies on the clinical efficacy of DNG maintenance treatment directly following conservative surgery in patients with endometriosis, especially in terms of recurrence, pregnancy rates, and related pain. However, its effects on long-term maintenance treatment after surgery need to be explored in detail. In addition, further verification should be conducted to comprehensively assess DNG as a maintenance treatment following conservative surgery for endometriosis.

Given the above background, the current study performed a systematic review and meta-analysis to comprehensively assess the value of DNG as a maintenance treatment for patients with endometriosis after conservative surgery. This was conducted with a particular focus on outcomes, such as disease recurrence, visual analog scale (VAS) scores, pregnancy rates, adverse effects, patient satisfaction, and CA125 levels.

## Materials and Methods

### Search Strategy and Study Design

The PubMed, Cochrane Library, Medline, and EMBASE databases along with the Web of Science were used to search for relevant studies and trials from 1980 to November 2020. The following keywords were used: “dienogest,” “endometriosis,” “endometrioma,” “endometrioses,” or “endometriomas.” This systematic review and meta-analysis of randomized controlled trials (RCTs) and cohort studies compared DNG maintenance treatment with other treatments in patients with endometriosis following conservative surgery. This meta-analysis was based on the Preferred Reporting Items for Systematic Reviews and Meta-Analyses (PRISMA).

### Inclusion Criteria

Two researchers read the title and abstract before reviewing the full text in detail. In addition, RCTs and prospective and retrospective studies conducted on humans were included if they met all of the following criteria: (1) premenopausal women undergoing conservative surgery for endometriosis; (2) at least two groups, including patients directly treated with DNG, were compared with those receiving other treatments, including LNG-IUS, GnRH-a, and non-treatment, separately, regardless of dosage, duration of treatment, and adverse effects. Patients who were not treated with DNG were considered controls; (3) at least one of the following outcomes: disease recurrence, VAS scores, pregnancy rates, adverse effects, patient satisfaction, and CA125 levels; (4) English language.

### Exclusion Criteria

The study excluded duplicates, reviews, comments, animal trials, case reports, abstracts, single-arm studies, unrelated topics, literature without full text, low-quality studies, and non-English literature. Thereafter, studies that were finally included were independently reviewed by two researchers, and all contradictions were settled by the third researcher.

### Measured Outcomes

The measured outcomes included: (1) the primary outcome, which in this case was disease recurrence and was defined as (i) radiographic recurrence of endometriosis (ultrasound) or (ii) symptom recurrence after receiving DNG maintenance treatment and in the control group not treated with DNG maintenance treatment, or (iii) the findings of the second laparoscopy. (2) The secondary outcomes included the VAS scores, pregnancy rates, adverse effects, patient satisfaction, and CA125 levels. In addition, symptom recurrence was defined as recurrence of subjective pain (dysmenorrhea, dyspareunia, or pelvic pain) and increased pain based on the VAS scores. Moreover, the adverse effects primarily included vaginal bleeding and weight gain. All outcomes should have been followed up for at least 12 weeks postoperatively with a maximum of 120 months.

### Quality Assessment and Data Extraction

The risk of bias for RCTs was assessed using the Modified Jadad scale in four domains: random sequence production, allocation concealment, blinding method, and withdrawal. Each item was classified as adequate, unclear, or inadequate in every domain. Notably, the Modified Jadad scale has a full score of 7, and 4–7 points are considered to be of high quality. Additionally, the Newcastle-Ottawa Scale was used for cohort trials, and it depends on three subscales: selection (four items), comparability (one item), and outcome (three items). A study could be awarded a maximum of one star for each numbered item within the selection and exposure categories. Moreover, a maximum of two stars could be assigned for comparability, and studies with six or more stars were considered to be of high quality, while those with five or fewer stars were regarded as low quality.

Additionally, two reviewers evaluated the quality of the included articles and extracted data, while the third reviewer discussed and resolved the conflicts. The contents of data extraction included the first author, date of publication, type of study, age, body mass index (BMI), number of patients, intervention measures, control measures, duration of treatment, and follow-up and outcome results.

### Data Synthesis

STATA software version 15.0 (STATA Corporation, College Station, TX) was used in this meta-analysis. Herein, the Q test (*I*^2^ value) was used to evaluate the heterogeneity of the included studies. Notably, a fixed-effects model was adopted if there was no significant heterogeneity (P > 0.1, when *I*^2^ ≤ 50%); otherwise, a random-effects model was used. In addition, the odds ratio (OR) was calculated in dichotomous data, while the standardized mean difference (SMD) with 95% confidence interval (CI) was computed for continuous values. Similarly, subgroup analyses were conducted according to specific interventions and follow-up time, and values of *p* < 0.05 were considered statistically significant.

## Results

### Selection and Characteristics of Studies

**Figure 1:**
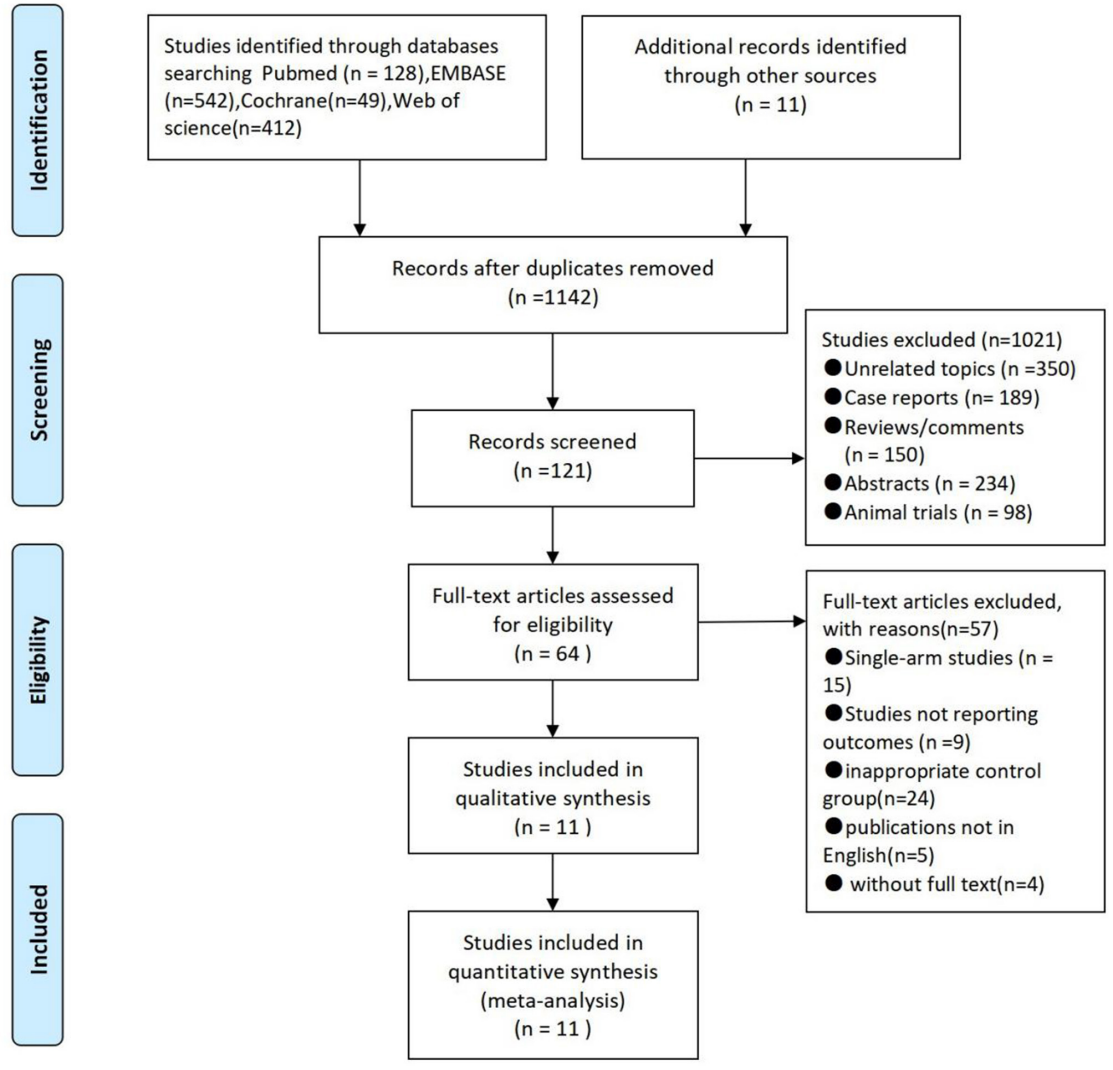
Flow diagram of study selection.

In total, 1,142 studies were searched from electronic databases. In total, 1,021 trials were excluded due to unrelated topics or because they were case reports, reviews, comments, abstracts, animal trials, or duplicates. The remaining 64 studies were subjected to further evaluation after which 57 were excluded because they were either single-arm studies or studies not reporting outcomes, had an inappropriate control group, were non-English literature, or literature without the full text. Finally, this meta-analysis included 11 studies with two RCTs comparing 198 patients and nine cohort trials comparing 1,323 patients. The characteristics of these 11 studies are shown in Figure 1 and Table 1 (7–17).

**Table 1:** Study characteristics.

	**References**	**Study design**	**Intervention**					**Control**					**Follow-up**	**Jadad/ NOS score**
			***n***	**Age (y) mean ± SD**	**BMI (kg/m^**2**^) mean ± SD**	**Types**	**Dosage and duration**	***n***	**Age (y) mean ± SD**	**BMI (kg/m^**2**^) mean ± SD**	**Types**	**Dosage and duration**		
1	Cosson et al. (7)	RCT	59	28.5 ± 4.9	58.4 ± 9.9	DNG	2 mg/day, 16 weeks	61	30.3 ± 5.1	57.2 ± 9.5	GnRH-a	3.75 mg/4 weeks, 16 weeks	16 weeks	5
2	Granese et al. (8)	RCT	39	31.2 ± 2.6	21.7 ± 2.3	DNG	2 mg/5 days; 3 mg/17 days, 9 months	39	30.5 ± 2.5	22.4 ± 2.1	GnRH-a	3.75 mg/30 days, 6 months	9 months	5
3	Lee et al. (9)	Retrospective	130	33.9 ± 6.1	20.8 ± 0.2	DNG	2 mg/day	72;83	43.7 ± 7.3; 39.3 ± 7.6	22.1 ± 3.2 21.5 ± 2.8	LNG-IUS; Non-treatment	LNG-IUS until pregnancy or premenopausal	>24 months	7
4	Takaesu et al. (10)	Prospective	54	32.4 ± 6.6	20.3 ± 2.0	DNG	2 mg/day, 24 weeks	51;79	35.9 ± 6.2; 34.6 ± 5.8	20.0 ± 2.020.7 ± 2.6	GnRH-a, non-treatment	1.8 mg/4 weeks, 6 times	24 months	6
5	Muller et al. (11)	Prospective	38	23-42	NA	DNG	2mg/day, 6 months	70;36	NA	NA	GnRH-a, non-treatment	3.75 mg/4 weeks, 6 times	6 months	6
6	Abdou et al. (12)	Prospective	121	29.52 ± 3.32	25.03 ± 1.45	DNG	2 mg/day, 12 weeks	121	29.77 ± 3.09	24.84 ± 1.47	GnRH-a	3.75 mg/4 weeks, 3 times	12 weeks	5
7	Yamanaka et al. (13)	Retrospective	59	35 ± 6.8	NA	DNG	2 mg/day	67	36 ± 5.9	NA	Non-treatment	NA	>6 months	7
8	Dobrokhotova et al. (14)	Retrospective	33	31 ± 2.5	NA	DNG	2 mg/day, 6 months	20	30.1.5	NA	Non-treatment	NA	12 months	6
9	Dong-Yun et al. (15)	Retrospective	36	29.0 ± 5.9	20.6 ± 3.1	DNG	2 mg/day, 6 months	28	30.6 ± 6.1	20.0 ± 3.0	GnRH-a	3.75 mg/4 weeks, 6 times. Estradiol: 1.0 mg/day. Norethisterone acetate0.5 mg/day	6 months	7
10	Morelli et al. (16)	Retrospective	48	33.42 ± 3.89	23.60 ± 2.44	DNG	one tablet/day	44	32.36 ± 3.25	24.09 ± 2.48	LNG-IUS	52 mg of levonorgestrel, releasing 20 mcg/24 h.	24 months	6
11	Ouchi et al. (17)	Retrospective	7	34.6 ± 5.8	NA	DNG	2 mg/day, 6 months	16 110	31.0 ± 5.5 34.9 ± 6.5	NA	GnRH-a, non-treatment	1.8 mg/4 weeks,6 times	120 months	6

*DNG, dienogest; LNG-IUS, levonorgestrel-releasing intrauterine system; GnRH-a, gonadotropin-releasing hormone analogs; NT, non-treatment; SD, standardized difference; BMI, body mass index; NA, Not applicable*.

### Assessment of Bias Risk

The risk of bias for the two included RCTs was assessed using the Modified Jadad scale in four domains. Random sequence production: two RCTs (7, 8) used an adequate method of random sequence production (computer randomization or random number tables). Allocation concealment: two RCTs (7, 8) provided information about the method of allocation concealment (the center or pharmacy controls the allocation plan, and the doctor and the subject cannot predict the allocation sequence). Blinding method: two RCTs (7, 8) lacked blinding of participants, and personnel was judged to generate detection bias (no double-blind or inappropriate blinding method). Withdrawal: two RCTs (7, 8) described the number and reasons for withdrawal. In contrast, the Newcastle-Ottawa Scale was used for cohort trials. All included studies were above 7 points, with a maximum of 9 points, as shown in Table 2.

**Table 2:** Assessment of bias risk of cohort studies.

**References**	**Newcastle-Ottawa scale**			
	**Selection**	**Comparability**	**Outcome**	**Total**
Lee et al. (9)	⋆⋆⋆⋆	⋆	⋆⋆⋆	8
Takaesu et al. (10)	⋆⋆⋆⋆	⋆⋆	⋆⋆⋆	9
Muller et al. (11)	⋆⋆⋆⋆	⋆	⋆⋆	7
Abdou et al. (12)	⋆⋆⋆⋆	⋆⋆	⋆⋆⋆	7
Yamanaka et al. (13)	⋆⋆⋆⋆	⋆⋆	⋆⋆	8
Dobrokhotova et al. (14)	⋆⋆⋆⋆	⋆	⋆⋆⋆	8
Lee et al. (15)	⋆⋆⋆⋆	⋆⋆	⋆⋆	8
Morelli et al. (16)	⋆⋆⋆⋆	⋆⋆	⋆⋆⋆	9
Ouchi et al. (17)	⋆⋆⋆⋆	⋆	⋆⋆	7

*⋆ represents one point in the Newcastle-Ottawa scale*.

### Effects of Intervention

All outcomes results are shown in Table 3.

**Table 3:** Comparison of DNG, OT, and NT in all outcomes.

**Categories**	**Number of studies**	**Number of patients**	**Model**	**OR/SMD (95% CI)**	***I*^**2**^ (%)**	***P_***h***_***	***Z***	***P***
**Disease recurrence**								
Total	9	1,081	Random	0.24 (0.15–0.37)	26.6	0.207	6.53	<0.001
DNG vs. other treatments	5	499	Random	0.46 (0.24–0.86)	<0.1	0.899	2.42	0.015
DNG vs. non-treatment	4	582	Random	0.14 (0.07–0.26)	<0.1	0.427	6.08	<0.001
**Three-month VAS scores**								
Total	4	137	Fixed	−0.35 (−1.09,0.40)	73.5	0.01	0.38	0.701
DNG vs. other treatments	1	74	Fixed	−0.38 (−0.08,0.84)	NA	NA	1.63	0.103
DNG vs. non-treatment	3	63	Random	−0.64 (−1.16, −0.12)	32	0.23	2.42	0.015
**12-month VAS scores**								
Total	6	549	Random	−1.20 (−2.15, −0.25)	95.1	<0.001	11.2	<0.001
DNG vs. other treatments	2	273	Random	−0.47 (−0.70, −0.23)	<0.1	0.416	3.82	<0.001
DNG vs. non-treatment	4	276	Random	−1.59 (−2.88, −0.31)	90.9	<0.001	13.13	<0.001
**Adverse effects**								
Total	2	358	Random	0.30 (0.03–2.70)	91.7	0.001	3.07	0.002
**DNG vs. other treatments**								
Vaginal bleeding	4	642	Random	8.71 (0.74–102.66)	94.7	<0.001	7.28	<0.001
Weight gain	2	306	Fixed	3.38 (1.14–9.98)	<0.1	0.829	2.20	0.028
**Pregnancy rates**								
Total	3	276	Fixed	1.96 (1.17–3.30)	13.5	0.315	2.56	0.011
DNG vs. other treatments	2	202	Fixed	1.55 (0.85–2.82)	<0.1	0.992	1.42	0.155
DNG vs. non-treatment	1	74	Fixed	4.05 (1.37–11.98)	NA	NA	2.53	0.012
**Patient satisfaction**								
DNG vs. other treatments	2	212	Random	0.50 (0.04–6.72)	80	0.025	0.52	0.605
Total	4	400	Random	−0.09 (−0.39–0.21)	54.7	0.085	0.60	0.548
DNG vs. other treatments	3	267	Random	−0.10 (−0.54–0.35)	69.8	0.037	0.43	0.667
DNG vs. non-treatment	1	133	Fixed	−0.09 (−0.43–0.26)	<0.1	NA	0.49	0.624

*A fixed-effects model was adopted if there was significant heterogeneity (P-value > 0.1, when I^2^ ≤ 50%); otherwise, a random-effects model was used*.

*vs, versus; OR, odds ratio; SMD, standardized mean difference; CI, confidence interval; DNG, dienogest; P_h_, P-value for heterogeneity based on Q test; P, P-value for statistical significance based on Z test; RCT, Randomized Controlled Trial; NA, not applicable*.

#### Disease Recurrence

**Figure 2:**
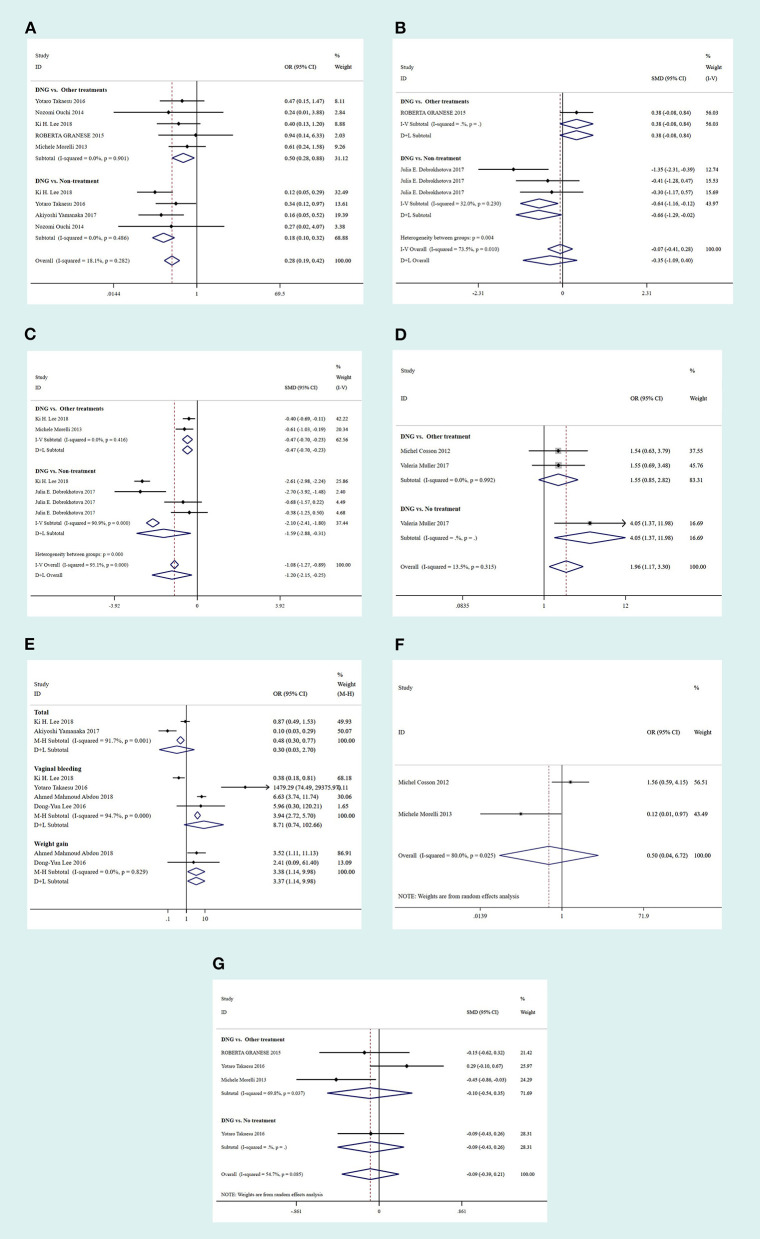
Forest plots of outcomes comparing the Dienogest (DNG) group with the control group in terms of disease recurrence **(A)**, 3-month VAS scores **(B)**, 12-month VAS scores **(C)**, adverse effects **(D)**, pregnancy rates **(E)**, patient satisfaction **(F)** and CA125 levels **(G)**. CI, confidence interval; OR, odds ratio; SMD, standardized mean difference.

This included nine studies, five of which compared DNG maintenance treatment to OT, while the remaining four compared DNG maintenance treatment to NT. The fixed-effects model was adopted because of a *p* > 0.1. Compared to OT or NT, DNG maintenance treatment significantly reduced disease recurrence in patients with endometriosis following conservative surgery (NT: OR 0.14, 95% CI: 0.07 to 0.26; *P* < 0.001; OT: OR 0.46, 95% CI: 0.24 to 0.86; *P* = 0.015). The results are shown in Figure 2A.

#### Three-Month VAS Scores

In total, four studies were included, three of which compared DNG maintenance treatment with NT, and the other one compared DNG maintenance treatment with OT. The fixed-effects model was similarly adopted because of a *p* > 0.1. The results showed that DNG maintenance treatment significantly decreased the VAS scores at 3 months postoperatively compared to NT (SMD: −0.64, 95% CI: −1.16 to −0.12; *P* = 0.015). However, no significant difference was observed between DNG maintenance treatment and OT (SMD: −0.38, 95% CI: −0.08 to 0.84; *P* = 0.103) from the fixed-effects model, as shown in Figure 2B.

#### Twelve-Month VAS Scores

In total, six studies were included, two of which compared treatment with DNG to OT, while the remaining four compared DNG maintenance treatment to NT. The random-effects model was adopted because of a *p* < 0.1. Notably, DNG maintenance treatment decreased the VAS scores at 12 months postoperatively, compared to both OT and NT (OT: SMD: −0.47, 95% CI: −0.7 to −0.23; *P* < 0.001; NT: SMD: −1.59, 95% CI: −2.88 to −0.31; *P* < 0.001), as shown in Figure 2C.

#### Pregnancy Rates

In total, three studies were included, two of which compared DNG maintenance treatment to OT while the remaining one compared DNG maintenance treatment to NT. The fixed-effects model was adopted because of a *p* > 0.1. Compared with NT, DNG maintenance management elicited a high pregnancy rate in patients with endometriosis following conservative surgery (OR: 4.05, 95% CI: 1.37 to 11.98; *P* = 0.012). However, no statistically significant difference was observed between DNG maintenance management and OT (OR: 1.55, 95% CI: 0.85 to 2.82; *P* = 0.155), as shown in Figure 2E.

#### Other Indices

In total, eight studies were included, four of which mentioned vaginal bleeding while two mentioned weight gain compared to OT. The findings revealed that DNG maintenance treatment significantly increased the overall adverse effects compared to OT (OR: 0.30, 95% CI: 0.03 to 2.70; *P* = 0.002), especially vaginal bleeding (OR: 8.71, 95% CI: 0.74 to 102.66; *P* < 0.001) and weight gain (OR: 3.38, 95% CI: 0.04 to 6.72; P = 0.028). Nevertheless, no significant difference was observed regarding patient satisfaction (OT: OR: 0.50, 95% CI: 1.14 to 9.98; *P* = 0.605) and CA125 levels (OT: SMD: −0.10, 95% CI: −0.54 to 0.35; *P* = 0.667; NT: SMD: −0.09, 95% CI: −0.43 to 0.26; *P* = 0.624), as shown in Figures 2D,F,G.

## Discussion

Most existing studies were designed to analyze the value of DNG in patients with endometriosis who had not undergone surgery. However, there is still inadequate research on DNG maintenance treatment postoperatively.

Zakhari et al. (18) indicated that DNG maintenance treatment following conservative surgery for endometriosis reduces the risk of disease recurrence compared to NT. However, the study did not compare DNG maintenance treatment with other treatments, such as LNG-IUS or GnRH-a; therefore, it gave no liberty to choose a suitable treatment according to patient needs. Moreover, Wattanayingcharoenchai et al. (19) suggested that there was no evidence supporting hormonal treatment for the postoperative prevention of endometriosis recurrence in the Network Meta-Analysis of RCTs. This was contrary to the evidence from cohort studies, which showed the protective effect of progesterone, especially for long-term management after surgery. Notably, both analyses mentioned the relationship between DNG maintenance treatment and disease recurrence postoperatively; however, they only focused on the disease recurrence outcome and ignored other outcomes, such as pregnancy. For infertile patients, the main purpose of postoperative maintenance treatment is to prevent disease recurrence and, more importantly, ensure a successful pregnancy. Simultaneously, the VAS scores, adverse effects, patient satisfaction, and CA125 levels were equally important in the maintenance treatment postoperatively. So far, there has not been a comprehensive systematic review and meta-analysis of DNG maintenance treatment for patients with endometriosis after conservative surgery. Therefore, the current meta-analysis primarily focused on the above mentioned outcomes important to patients, which included disease recurrence and pregnancy rates, with the direct use of DNG as a maintenance treatment following surgery.

DNG moderately inhibits the H-P-O axis, thereby inhibiting estrogen production (19). Similarly, it downregulates prostaglandin E2, inflammatory cytokines [including interleukin (IL)-6, IL-8, monocyte chemoattractant protein-1], estrogen synthetase aromatase, and neuroangiogenesis factors (such as vascular endothelial growth factor (VEGF) and nerve growth factor) in endometriotic cells to decrease recurrence and alleviate pain (19, 20).

In this meta-analysis, 11 trials (two RCTs, six retrospective cohorts, and three prospective cohorts) consisting of 1,521 patients were included. Of the six retrospective cohorts, three used almost similar criteria radiologically through US or MRI, while two defined endometriosis recurrence as ≥ 2 cm of endometrioma either through US or MRI. The remaining study did not specify the criteria used. Furthermore, two studies defined recurrence not only through imaging but also through symptoms, such as dysmenorrhea, dyspareunia, and pelvic pain. Therefore, it is appropriate to incorporate the patient symptoms in the definition of disease recurrence; otherwise, some victims with recurrence of endometriosis may be omitted. Additionally, the physical examinations and laparoscopy findings should be incorporated in the future for a more accurate assessment of DNG maintenance treatment following conservative surgery for endometriosis.

In this analysis, DNG maintenance treatment significantly decreased disease recurrence compared to OT or NT, thus preventing reoperation. Compared with LNG-IUS treatment, DNG maintenance treatment caused a significant decrease in the VAS scores at 12 months postoperatively. These findings highlight the effectiveness of DNG as a maintenance treatment strategy postoperatively. Unfortunately, these were retrospective studies with small sample sizes; therefore, more samples are needed in the future.

Additionally, compared with OT, DNG maintenance treatment has no obvious advantage in increasing pregnancy rates. In addition, only three studies followed up pregnancy rates, and the sample size was small. Therefore, more studies with larger sample sizes following up pregnancy rates are needed in the future.

Moreover, DNG maintenance treatment caused a marked increase in the risk of vaginal bleeding and weight gain after conservative surgery. Herein, a total of eight studies were included, four of which separately mentioned vaginal bleeding, while the other four separately mentioned weight gain. The remaining two studies summarized the overall incidence of adverse effects, including loss of bone density and insomnia, hot flashes, and mood disorders. Therefore, the analysis showed that DNG, like progesterone, could increase vaginal bleeding in patients, and this has always been mentioned by both doctors and patients. However, the lack of a significant difference in patient satisfaction between DNG maintenance treatment and OT is probably because symptoms, such as vaginal bleeding or weight gain, are more acceptable than GnRH-induced symptoms, including osteoporosis, insomnia, hot flashes, and night sweats or those associated with LNG-IUS, such as abdominal pain, ring incarceration, ring pregnancy, and discomfort. Moreover, no significant difference in the CA125 levels was observed between the DNG treatment group and the control group. In view of the fact that the relationship between CA125 levels pre- and post-treatment and the prediction of recurrence or malignant transformation of endometriosis is still uncertain. Future researches can try to monitor the CA125 levels or other carbohydrate antigens pre- and post-treatment of endometriosis, and observe the disease outcomes.

DNG, as a postoperative maintenance treatment, significantly decreased the rates of disease recurrence. Nevertheless, it conferred no advantage over the other treatments in increasing pregnancy rates in women with infertility. However, it significantly increased the incidence of vaginal bleeding and weight gain. In conclusion, for women who have no fertility requirements, DNG seems to be a better choice because it can significantly decrease recurrence compared to OT. These findings will help patients choose suitable maintenance treatment after conservative surgery.

The current meta-analysis included two RCTs and nine cohort studies, meaning that the number of RCTs was small, while the retrospective cohort studies were biased. Although the 11 included articles were defined as good quality, the sample size of these articles was rather small, that is, 11 studies with two RCTs and nine cohort trials comparing 198 and 1,323 patients, respectively. In general, more RCTs or large-scale trials are needed to compare the DNG maintenance treatment of patients with endometriosis postoperatively with OT, classify the postoperative disease recurrence time, set different duration of treatment, and extend the follow-up time for pregnancy outcomes.

## Conclusions

DNG can be recommended as a maintenance treatment for patients with endometriosis to decrease the rates of disease recurrence following conservative surgery. However, DNG maintenance treatment has no advantage in improving pregnancy rate compared to OT. It is worth discussing in the future whether we can continue to use DNG maintenance treatment to further reduce the rates of disease recurrence and increase the pregnancy rates after using GnRH-a treatment more than six times postoperatively. Therefore, well-designed, large-scale prospective studies are still required to confirm the true benefit of DNG maintenance treatment.

## Data Availability Statement

The original contributions presented in the study are included in the article/supplementary material, further inquiries can be directed to the corresponding author/s.

## Author Contributions

YL and ZL designed the study. YL and JG searched the literature. YL and HG performed the statistical analyses. YL and XL drafted the manuscript. ZL revised important academic content. All authors interpreted the results, read and approved the final manuscript.

## Conflict of Interest

The authors declare that the research was conducted in the absence of any commercial or financial relationships that could be construed as a potential conflict of interest.
